# miREvo: an integrative microRNA evolutionary analysis platform for next-generation sequencing experiments

**DOI:** 10.1186/1471-2105-13-140

**Published:** 2012-06-21

**Authors:** Ming Wen, Yang Shen, Suhua Shi, Tian Tang

**Affiliations:** 1 State Key Laboratory of Biocontrol, School of Life Sciences, Sun Yat-sen University, Guangzhou, 510275, Guangdong, People’s Republic of China; 2 The Key Laboratory of Gene Engineering of Ministry of Education, School of Life Sciences, Sun Yat-sen University, Guangzhou, 510275, Guangdong, People’s Republic of China; 3 Guangdong Key Laboratory of Plant Resources, Sun Yat-sen University, Guangzhou, 510275, Guangdong, People’s Republic of China

## Abstract

**Background:**

MicroRNAs (miRNAs) are small (~19-24nt) non-coding RNAs that play important roles in various biological processes. To date, the next-generation sequencing (NGS) technology has been widely used to discover miRNAs in plants and animals. Although evolutionary analysis is important to reveal the functional dynamics of miRNAs, few computational tools have been developed to analyze the evolution of miRNA sequence and expression across species, especially the newly emerged ones,

**Results:**

We developed miREvo, an integrated software platform with a graphical user interface (GUI), to process deep-sequencing data of small RNAs and to analyze miRNA sequence and expression evolution based on the multiple-species whole genome alignments (WGAs). Three major features are provided by miREvo: (i) to identify novel miRNAs in both plants and animals, based on a modified miRDeep algorithm, (ii) to detect miRNA homologs and measure their pairwise evolutionary distances among multiple species based on a WGA, and (iii) to profile miRNA expression abundances and analyze expression divergence across multiple species (small RNA libraries). Moreover, we demonstrated the utility of miREvo with Illumina data sets from *Drosophila melanogaster* and *Arabidopsis*, respectively.

**Conclusion:**

This work presents an integrated pipline, miREvo, for exploring the expressional and evolutionary dynamics of miRNAs across multiple species. MiREvo is standalone, modular, and freely available at http://evolution.sysu.edu.cn/software/mirevo.htm under the GNU/GPL license.

## Background

MicroRNAs (miRNAs) are small non-coding RNAs about 19–24 nt in length that regulate gene expression post-transcriptionally [1,2]. MiRNAs are ubiquitous in eukaryotes and take part in the regulation of various key developmental events[3]. Extensive studies have been done, both experimental and bioinformatics, to unravel the mechanisms and characteristics of miRNA [4,5]. An important characteristic of miRNA is the high sequence conservation observed within either plant or metazoan kingdom. Some miRNAs are at least 400 million years old and even many miRNA:target interactions are broadly conserved [3,6]. In fact, the strong phylogenetic conservation has been a wildly-used criterion for the identification of miRNAs, such as in Arabidopsis thaliana [7] and human [8]. The observed conservation of miRNA between different species suggests that strong purifying selection is acting to maintain the miRNA genes. Despite the similarity of high conservation within kingdom, plant and animal miRNAs are different in many ways, including the biogenesis pathway, hairpin structures and the base-pairing model with their target sites [3,5]. These different properties has resulted that the miRNA analysis pipelines, such as novel miRNA prediction and target prediction, are quite different between plants and animals [9-11].

In the last few years, the next-generation sequencing (NGS), an inexpensive and high-throughput sequencing method with high sensitivity and specificity, has been widely used to measure the abundance of small-RNAs in multiple species [12-15]. Additionally, NGS can also be adopted to discover novel miRNAs [15,16]. To take the advantage of NGS, numerous applications, either web-servers or standalone, have been developed to analyze the deep sequencing data of small RNAs for miRNA discovery and/or expression profiling [4,9,17-22] (Table 1). Among these tools, miRDeep [15,22] is the most popular one, due to its great accuracy and performance. These new experimental and analysis techniques greatly accelerate the miRNA studies. To date, there are more than 18,200 reported miRNAs from 168 species in the miRBase database (http://www.mirbase.org, Version 18.0).

**Table 1:** Comparison of computational tools for microRNA analysis

**Feature**	**miROrtho**[4]	**miRNA-miner**[21]	**microHAR-VESTER**[20]	**miRanalyzer**[18]	**miRExpress**[17]	**miRDeep-P**[9]	**miRDeep2**[22]	**UEA sRNA toolkit**[19]	**miREvo**
Next-generation sequencing technology support	×	×	×	√	√	√	√	√	√
Expression profiling	×	×	×	√	√	×	√	√	√
comparison of orthologous gene expression	×	×	×	×	×	×	×	×	√
Novel miRNA Prediction for plant/animal	Animal	Animal	×	Both	×	Plant	Animal	Both	Both
Stem-loop structure viewing	√	√	√	√	√	√	√	√	√
Homology searching in plant/animal kingdom	Animal	Animal	Plant	×	×	×	×	×	Both
evolutionary distance calculating	×	×	×	×	×	×	×	×	√
Multiple miRNAs queries support	×	×	5 sequences max	×	×	×	×	√	√
Standalone/Web-service	Web	Web	Web	Both	Standalone	Standalone	Standalone	Both	Standalone
GUI/CLI	GUI	GUI	GUI	Both	CLI	CLI	CLI	GUI	Both

√, with the feature; ×, without the feature; GUI, Graphical User Interface; CLI, Command Line Interface.

Besides the conserved miRNAs, NGS has identified many new miRNA genes, which are highly divergent between closely-related species, such as miR310 cluster in *Drosophila*[23,24]. New miRNA genes are shown to have a high birth and death rate [25] and may represent the source of functional novelty [26]. Further evolutionary studies are required to illustrate the phylogenetic relationship and potential functions of these new miRNAs. Nevertheless, few analysis tools are designed for miRNA evolutionary study. Most of the available miRNA analysis tools are web-server and BLAST-searching based [20], which have limitations in screening highly divergent orthologs even with a stringent E-value cut-off. Moreover, many tools are specifically designed for animals [4,21] and thus less applicable for the evolutionary analysis of plant miRNAs, which have underwent extensive gene duplications and are usually dispersed in the genome as divergent family members with various copy number across species.

Meanwhile, whole genome sequencing has been completed for a great number of species. The multiple-species whole genome alignments (WGAs) provides us a luxuriant resource for comparative genomic analysis and evolutionary studies [27-29], and also facilitates the genome-wide studies on the miRNA evolutionary pattern globally [30]. For example, the WGA-based approach has been successfully used to reveal the highly dynamic evolutionary flux of miRNAs in both *Drosophila*[25] and *Arabidopsis*[16]. Such investigations enable us to understand the history of miRNAs in long term evolution, e.g., how a young miRNA is born and eventually integrates into the regulatory network [24,25], and how selection acts to maintain miRNA conservation or drive miRNA diversification [23].

Here, we reported miREvo, an integrated miRNA evolutionary analysis platform for NGS experiments by using miRDeep2 [22] as core algorithm in miRNA prediction. miREvo is readily to use for both plant and animal miRNA analysis, including miRNA expression evaluation, novel miRNA prediction, miRNA homology search and between-species comparative analysis based on WGA. miREvo is a flexible, GUI supported, standalone package; we believe it will provide a convenient aid to miRNA research community.

## Implementation

### User interface overview

**Figure 1:**
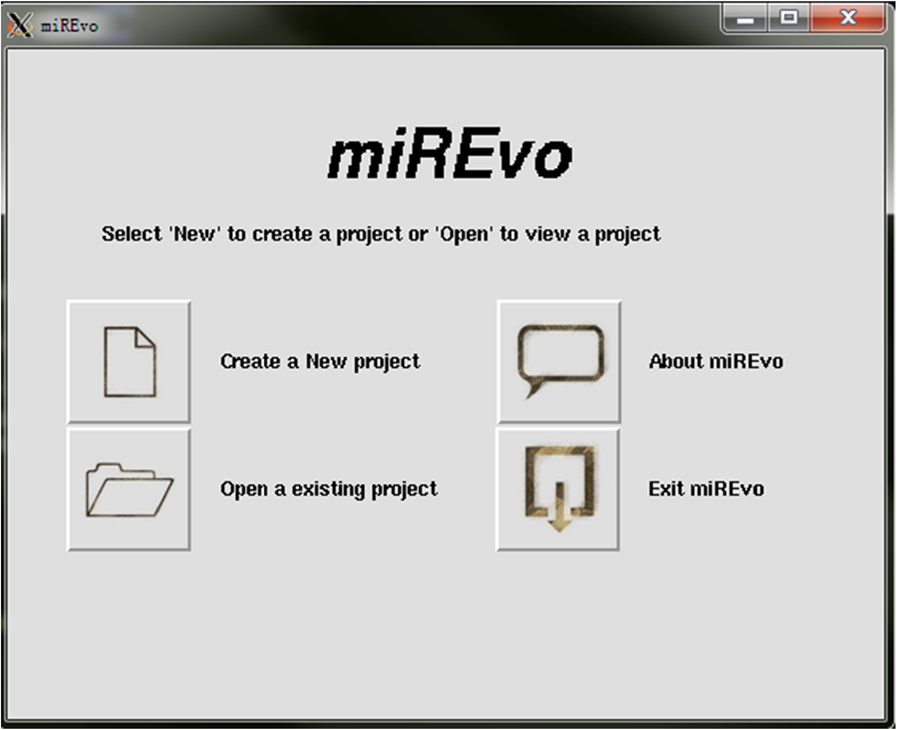
**Initial interface of miREvo.** Once the GUI is launched, users can open an existing project or create a new project in miREvo.

MiREvo is committed to provide an efficient and easy-to-use solution for miRNA analysis based on NGS experiments. To achieve this goal, we have implemented miREvo with both command-line interface (CLI) and Graphical User Interface (GUI, Figure 1). The GUI is an interactive application used to create miRNA projects, specify project parameters, run the computer algorithms on the project data, and view the output of computations lively. Under the GUI, users can easily view, control and manipulate miREvo with a few mouse clicks at once (Additional file 1: Figure S1). On the other hand, The CLI is used to deal with heavy tasks with multiple small RNA libraries.

### Software architecture

**Figure 2:**
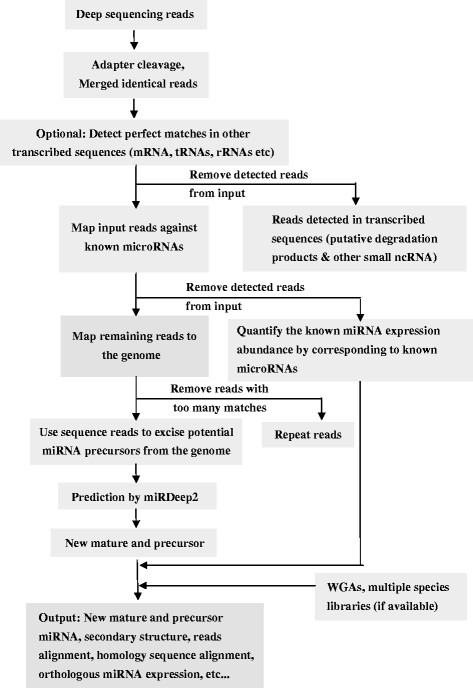
**System flow of the construction of miRNA expression profiles in miREvo.** miREvo accepts second-generation sequencing data as input and then can generate miRNA expression profiles, predict novel miRNAs and identify homology sequence of miRNAs.

The system flow of implementation used in miREvo is simplified in Figure 2, which accepts NGS data as input. Typically, for a full analysis run, the following first three analyses are carried out to generate the final results. When NGS data from multiple small RNA libraries are provided, miREvo is also capable to compare miRNA expression profiles between libraries, as described in the last section here.

#### Preparing the deep-sequencing reads

After removing the adapter at the 3’ends of sequencing reads, the identical reads are collapsed into a unique read and counted. Then, all the unique reads are aligned to the corresponding reference sequences by Bowtie [31] in each step. The Bowtie parameters used for reads mapping are almost identical to the settings of miRDeep2. Firstly, reads are aligned (up to two mismatches) to a user defined database that contains annotated rRNA, small cytoplasmic RNA (scRNA), small nuclear RNA (snRNA), small nucleolar RNA (snoRNA), and tRNA or protein coding regions. This is done with these options: bowtie -f -v $MIS -a --best --strata. The –v option is used to retain reads that have no more than $MIS mismatch of a hits to the genome, where $MIS is a user-specified integer value. These aligned reads could be discarded from further analysis. Next, the remaining reads are aligned (bowtie -f -v 1 -a --best --strata --norc) to the precursors of known miRNAs if provided, to detect the expression abundance of these miRNAs. Lastly, after excluding the reads mapped to the known miRNAs, the remaining reads are used to predict novel miRNAs (Figure 2).

#### Predicting the novel miRNAs

miREvo uses miRDeep2 [22], a freely software package developed by Max Delbrück Center, to identify novel miRNAs from deep sequencing data. MirDeep is a probabilistic-model-based method for miRNA discovery in animals, which has been demonstrated to be sensitive and accurate in several studies [32-34]. For animals, the default parameters of miRDeep2 are used in miREvo. To extend miRDeep2 for plant miRNA prediction, parameters are well-adjusted according to the statistical characteristics of plant miRNAs [5,9,11]. For instance, more repeated matching is allowed in reads alignment step since plant miRNAs typically possess larger families than the animals. Other features including minimum free energy, stability of secondary structures, and excision length, etc., are also restricted as described previously [3], these new parameters are listed in Additional file 2. To further refine the prediction of novel miRNAs, miREvo provides a new feature of crosschecking with known miRNAs from species of interests in miRBase. Known miRNAs with identical seed and/or mature sequence more than 80% identity with the predicted new one will be reported.

#### Identifying the miRNA homologs

One challenge in homology identification is to produce a single best orthologous match for a given miRNA/sequence. To reduce the interference by tandem duplication sequences, WGA is well-adjusted and optimized for orthologous alignment extension and specificity [35]. miREvo uses a reference guide method to locate the homologous sequences among the WGA between relative species. Firstly, miRNA precursors are precisely mapped to the reference using BLAT. Next, the whole homologous region is excised from the Multiple Alignments File (MAF) using mafsInRegion (http://genomewiki.ucsc.edu/index.php/Kent_source_utilities), according the coordinate of the miRNA precursor located in the reference. Compared with the methods that directly align miRNAs against the reference genome using BLASTN, our WGA-based method is more credible in screening the orthologs of highly divergent miRNAs.

To access MAF files, UCSC (http://genome.ucsc.edu) has built multiple genome alignments for most sequenced animals, such as vertebrates or inserts. For the plants, we have constructed two MAF files. One represents the alignment of 11 green plant genomes (Additional file 1: Table S1) using *Arabidopsis thaliana* genome as the reference. The other is a pairwised alignment between rice (*Oryza sativa*) genomes of Japonica cv. *Nipponbare* and Indica cv. 93–11 (Additional file 1: Table S1), using Nipponbare genome as the reference. Multiple alignments with additional genomes will be regularly added in further release versions. These two MAF files were generated by using BLASTZ [35] and other tools from the UCSC/Penn State Bioinformatics comparative genomics alignment pipeline [35-37]. Briefly, plant genome sequences were downloaded from Phytozome (http://www.phytozome.net/, version 7.0) or NCBI (see Additional file 1: Table S1 for the full list of aligned species). After repeat masking by Tandem Repeat Finder [38], all the genomes were aligned to the reference genome, e,g. *Arabidopsis thaliana* genome, using BLASTZ; Two matching alignments next to each other were joined into one fragment using axtChain [37]; Blocks of chained alignments were further grouped into longer stretches of synteny using netChain [37]. Finally, the sequences were retrieved from the synteny-files and the alignments were re-created.

#### Comparing orthologous miRNA expression

Once the orthologs are obtained, a consequent desire would be to compare the expression divergence of miRNAs across different species of interests. To fulfill this need, we further supplied a simple solution to support cross-species comparison of miRNA expression. The orthologous sequences from each species are extracted from the WGA according to their coordinates, and then used as reference for short reads mapping in each species with Bowtie (bowtie -f -v 1 -a --best --strata --norc). The expression of mature miRNAs from 5’ and 3’ arms of the hairpin precursors is estimated by counting the mapped reads and normalized by the total number of mapped reads, respectively. Then, a Poisson regression model [39] and likelihood ratio test are employed to obtain the statistical significance of differential expression between species. The final results are presented as a table of homologous miRNA expression and a scatter plot.

## Results

### Identification of novel miRNAs

**Figure 3:**
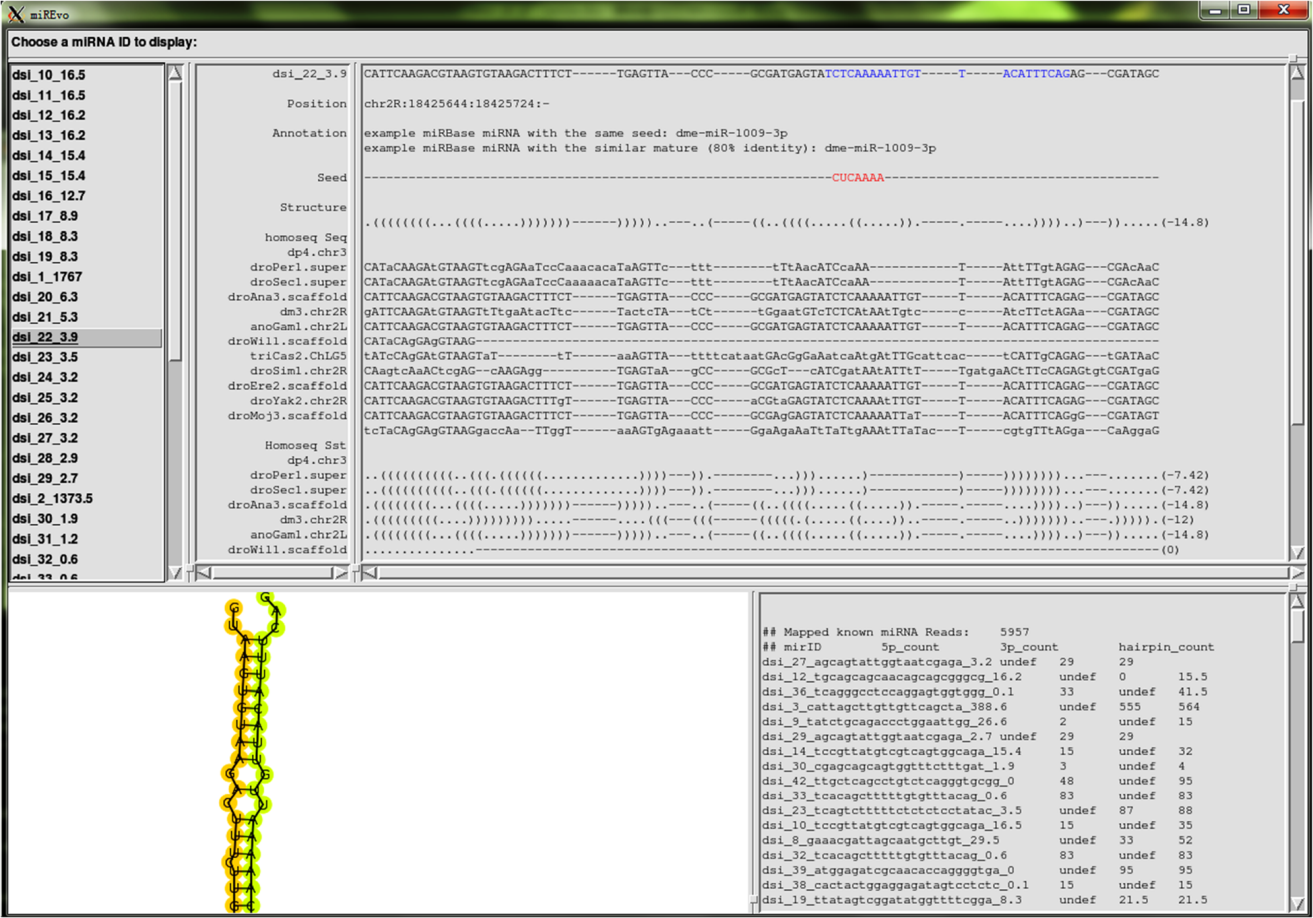
**Overview of the result viewing interface of miREvo.** The miREvo result presenting interface contains four panels: A, miRNA list table; B, result navigator; C, hairpin structure viewing; and D, miRNA expression table.

To illustrate the usage of miREvo, we applied miREvo to two small RNA NGS datasets, which were retrieved from Gene Expression Omnibus (GEO) database (http://www.ncbi.nlm.nih.gov/geo/). One was from *Drosophila simulans* embryo (GSM343915) and the other was from *Arabidopsis thaliana* leaves (GSM707679). As a working example, we only presented the results of *Drosophila melanogaster* here. The example of *Arabidopsis thaliana* was provided in the additional files. The full demo data can be downloaded from our website (http://evolution.sysu.edu.cn/software/mirevo.htm). After a full run of analysis, we provided the following results (Figure 3):

(1) Reads Statistics of miRNAs: A total of 4,609,104 reads were sequenced, representing 863,547 unique signatures. A table summarized the reads count for each miRNA was provided. Among the 136 characterized miRNA genes of *D. simulans* (miRbase V18.0), 101 were observed with a total of 1,746,656 reads (11,538 unique signatures) mapped to the corresponding loci. In addition, another 43 putative new miRNAs with 5,957expressed reads were predicted using default criteria of miRDeep2[32]. Among them, 13 have hits in other *Drosophila* species by crosschecking with miRBase. For example, the novel miRNA dsi_22_3.9 has been annotated as miR-1009 in *D. melanogaster* (Figure 3). Full reports, including Html format report generated by miRDeep2, were retained under the user specified project folder.

(2) FASTA sequences and multiple alignments: The orthologous sequences of the predicted new miRNAs in the other 11 *Drosophila* species were parsed out from the 12 *Drosophila* species WGA produced by UCSC (http://hgdownload.cse.ucsc.edu/goldenPath/dm3/multiz15way/). A multiple alignment of these orthologous sequences were constructed. To evaluate the RNA secondary structure conservation, the alignments of minimum free energy (mfe) structure were also displayed in bracket notation. Finally, the pairwise genetic distances (Kmir, based on the Kimura two-parameter model [23]) and sequence identity of miRNA homologs were calculated for precursor, mature and seed regions, separately.

(3) The minimum energy and the miRNA hairpin folding. The hairpin folding of individual pre-miRNA was graphed with detailed information of folding free energy. The pre-miRNA sequence was underlined as color-coded according to the reads’ coverage pre base of the hairpin.

### Identification of homologous miRNAs

Another application of miREvo is to identify the homologous sequences of a given miRNA. To do so, MiREvo requires input files including 1) the precursor miRNA sequence, 2) the mature miRNA sequence, 3) the reference genome, and 4) the MAF files containing the WGA of species of interests; the output file presents the best-fitted results. Taking the advantage of synteny information of WGA, miREvo is particularly useful in identifying orthologs of highly divergent miRNAs, or paralogous members of old miRNA families. To demonstrate that the WGA-based method has better performance than BLASTN, we conducted a genome-wide screening for all the orthologs of the 240 known miRNA of *D.melanogaster* (miRBase V18) in genome of *D.pseudoobscura* using both methods. Considering miRNA precursors that have at least 90% identity out of a coverage length greater than 50nt between the two species, miREvo identified 93 (39%) orthologs with at least ten sequencing reads at the embryonic stage in *D. pseudoobscura*. By contrast, the BLASTN-based search (Options: -e 0.01 -W 7 -b 3 -v 3 -q −1 -F F) only identified 66 (28%) orthologs. Therefore, the WGA-based method is significantly more effective (p value = 0.012, chi-square test).

**Figure 4:**
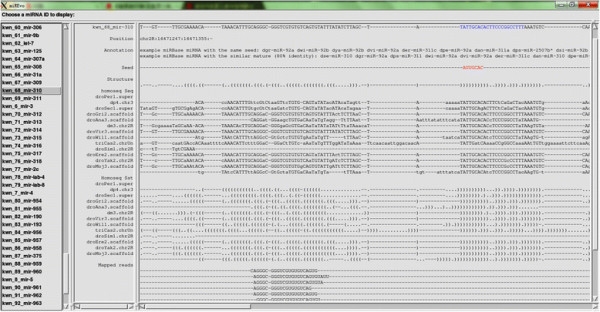
**Othologs of dme-miR-310 across 12*Drosophila*genome as identified by miREvo.**

As a case in point, miR-310 is rapidly evolving in *Drosophila*, which has accumulated a large number of nucleotide substitutions since the split of *D. melanogaster* and *D. pseudoobscura*. The orthologous sequence of dme-miR-310 in *D. pseudoobscura* can be clearly identified with miREvo (Figure 4). By contrast, a BLASTN [40] searching for the same miRNA with word size 7nt generated no hit in *D. pseudoobscura.* The evolutionary distance (Kmir) of dme-miR-310 between the two species is 0.5, which is half of the averaged genome-wide synonymous divergence [41]. The Kmir/Ks ratio less than 1 suggests that dme-miR-310 is under functional constrain although it might have evolved differentially between species. A more complex case of the homology identification of *Arabidopsis* ath-miR-169 family was given in Additional file 3. Using miREvo, orthologs for all the members of *Arabidopsis* ath-miR-169 family can be individually distinguished (Additional file 3: Figure S2).

### Expression profiling of orthologous miRNAs

**Figure 5:**
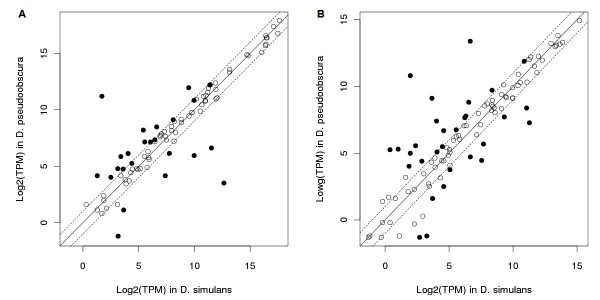
**Differential expression orthologous miRNAs between*D. simulans*and*D. pseudoobscura.*** The expression of mature miRNAs from 5’ (**A**) and 3’ (**B**) arms of the hairpin precursors were estimated separately. The normorlized expression levels of miRNAs (Log2 TPM) in *D. pseudoobscura* (y-axes) were plotted against those of their orhologs in *D. simulans* (x-axes). MiRNAs that differentially expressed between species or not were indicated as solid or open circles, respectively. Dsi, *D. simulans*; Dps, *D. pseudoobscura*; TPM, Tags per million mapped reads.

To demonstrate the utility of miREvo in cross-species comparison of miRNA ortholog expression in an identical cell type, we applied it to two published libraries generated from *Drosophila* species:*D. simulans* (GSM343915) and *D. pseudoobscura* (GSM343916). Using *D. melanogaster* miRNAs as references, orthologs from the other two species were extracted. The expression of orthologs for each library was analyzed separately, cross-species expression profiles were constructed (Additional file 1: Table S2). Out of 161 orthologous miRNA genes, statistical analysis indentified 47 and 42 mature miRNAs from 5’ and 3’ arms of the hairpin precursors, respectively, that are differentially expressed between *D. simulans* and *D. pseudoobscura* (Figure 5 A and B). In summary, miREvo offers the capacity to identify the orthologous miRNAs and also to compare the expression differences among orthologs between species. The demo data and scripts described here can be downloaded at miREvo web site: http://evolution.sysu.edu.cn/software/dro_orth_exp.tar.gz.

## Conclusions

In this report, we introduced miREvo, an integrated platform for miRNA NGS data analysis. miREvo enables batch annotation of the small RNA reads generated by NGS. It also provides flexibility with the tight integration of the miRDeep package, which enables users to predict novel miRNAs for both plants and animals. The main advantage of miREvo is the ability/flexibility to identify miRNA orthologs in closely or distantly related species based on WGA, which is superior to the blast-searching-based tools [4,20,26]. To accelerate the evolutionary study of plant miRNAs, we have provided two build-in WGA datasets among multiple plant species. To our knowledge, it is the first available tool for evolutionary analysis of miRNA deep sequencing dataset for both plants and animals. In addition, miREvo is standalone and can be accessed via a GUI or from a CLI. We think miREvo will be a great convenience and facilitate the downstream analysis of miRNA sequencing data.

## Availability and requirements

· **Project name:** miREvo

· **Project home page**: http://evolution.sysu.edu.cn/software/mirevo.htm

· **Operating system(s):** Unix/Linux based

· **Programming language**: Bash, Perl 5 and Perl/TK

· **Other requirements:** RNAfold, Bowtie, BioPerl, mafsInRegion and BLAT

· **License:** GNU GPL v3

· **Any restrictions to use by non-academics:** specified by GNU GPL v3

Before the application can be started, the following open source packages must be installed:

1. Perl 5 – http://www.perl.org/get.html

2. Perl-TK – http://search.cpan.org/~ni-s/Tk-804.027/pod/UserGuide.pod

3. BiolPerl – http://www.bioperl.org/wiki/Getting_BioPerl

4. The following free available packages are also needed by miREvo, which has been deposited into miREvo package, or users can install them independently:

(a) Vienna RNA Package – http://www.tbi.univie.ac.at/~ivo/RNA/

(b) Bowtie – http://bowtie-bio.sourceforge.net/index.shtml

(c) masfsInRegion – http://genomewiki.ucsc.edu/index.php/Kent_source_utilities

(d) BLAT – http://genome.ucsc.edu/FAQ/FAQblat.html#blat3

e) ImageMagick (http://www.imagemagick.org/script/index.php)

Such information is given in the user manual accompanied with miREvo. All these packages can also be downloaded from http://evolution.sysu.edu.cn/software/utilities.tar.gz. The documentation also illustrates the usage of each individual functions, including the case-oriented help session that demonstrate the examples described in the paper.

## Abbreviations

miRNA, microRNA; NGS, Next-generation sequencing; WGA, whole genome alignment.

## Competing interests

The authors declare that they have no competing interests.

## Authors' contributions

MW and YS developed miREvo and carried out the analysis. YS and TT conceived and directed the project. MW, YS, SSH and TT wrote the manuscript. All authors have read and approved the final manuscript.

## Supplementary Material

Additional file 1 **Supplementary Materials.doc**. The Supplementary Material for the paper. Click here for file

Additional file 2 **Plant Parameters.doc**. The modification of miRDeep2 parameters that are used for the prediction of novel plant miRNAs in miREvo. Click here for file

Additional file 3 **Supplementary Figure S2.pdf**. Supplementary Figure S2, which is too large to include in the text. Click here for file
