# Supplementary material for: miREvo: an integrative microRNA evolutionary analysis platform for next-generation sequencing experiments
Source: BMC Bioinformatics. 2012 Jun 21;13:140. doi: 10.1186/1471-2105-13-140 (PMC3410788; doi:10.1186/1471-2105-13-140)
Supplement: Additional file 1 — Supplementary Materials.doc. The Supplementary Material for the paper. [file 1471-2105-13-140-S1.doc]

# Supplementary Materials

## Table of Contents

Supplementary Tables 2

Supplementary Figure Legends 6

Supplementary Figures 7

# Supplementary Tables

## Table S1 - The resources of plant genome datasets used for MAF construction.

| **Species** | **Code** | **Source** |
| --- | --- | --- |
| *Arabidopsis lyrata* | araLyr1 | [JGI release v1.0](http://genome.jgi-psf.org/Araly1/Araly1.info.html) |
| *Arabidopsis thaliana* | araTHA8 | [TAIR release 8 acquired from TAIR](http://arabidopsis.org/) |
| *Citrus clementina* | cirCle1 | [JGI v0.9 assembly and annotation](http://www.phytozome.net/clementine.php) |
| *Glycine max* | glyMax1 | [JGI Glyma1.0 annotation of the chromosome-based Glyma1 assembly](http://www.phytozome.net/soybean.php) |
| *Mimulus guttatus* | mimGut1 | [JGI 7x assembly release v1.0 of strain IM62, annotation v1.0](http://www.phytozome.net/mimulus.php) |
| *Oryza sativa ssp. indica (9311)* | osaInd1 | BGI release of [Superscaffold of Beijing indica](http://rice.genomics.org.cn/rice/statistics/jsp/SuperScaffold/SuperScaffoldTotal.jsp?organism=9311) |
| *Oryza sativa ssp. japonica* | osaJap1 | [MSU Release 6.0 of the Rice Genome Annotation](http://rice.plantbiology.msu.edu/) |
| *Physcomitrella patens ssp. patens* | phyPat1 | [JGI assembly release v1.1](http://genome.jgi-psf.org/Phypa1_1/Phypa1_1.info.html) |
| *Populus trichocarpa* | popTri1 | [NCBI assembly release: Poptr1_1](http://www.ncbi.nlm.nih.gov/genome/98) |
| *Selaginella moellendorffii* | selMoe1 | [JGI v1.0 assembly and annotation](http://genome.jgi-psf.org/Selmo1/Selmo1.info.html) |
| *Sorghum bicolor* | sorBio1 | [Sbi1.4](http://www.phytozome.net/sorghum) models from MIPS/PASA on v1.0 assembly |
| *Vitis vinifera* | vitVin1 | [March 2010 12X assembly and annotation](http://www.genoscope.cns.fr/externe/Download/Projets/Projet_ML/data/12X/) from Genoscope |

## Table S2 - Expression divergence of miRNA orthologs between *D. simulans* and *D. psuedoobscura*.

A total of 161 *D. melanogaster* miRNAs with orthologs in *D. simulans* and *D. psuedoobscura* were analyzed and the expression profiles were used for the statistical analysis in Figure 5. Dsi, *D. simulans*, Dps, *D. psuedoobscura*. TPM, TPM, Tags per Million mapped reads.

| mirna ID | Dsi Reads Count | Dps Reads Count | Dsi TPM | Dps TPM | Log2 fold_change | P value | Adjust P value |
| --- | --- | --- | --- | --- | --- | --- | --- |
| mir-969 3p | 6 | 5 | 2.44 | 2.18 | 0.12 | 0.88 | 1.00 |
| mir-970 3p | 1030 | 1380 | 449.00 | 561.00 | -0.52 | 0.00 | 0.00 |
| mir-971 3p | 0 | 139 | 0.00 | 60.59 | -7.23 | 0.00 | 0.00 |
| mir-6-2 3p | 70 | 64 | 30.51 | 26.02 | 0.03 | 0.91 | 1.00 |
| mir-980 3p | 604 | 660 | 245.54 | 287.71 | -0.23 | 0.00 | 0.26 |
| mir-981 3p | 200 | 400 | 87.19 | 162.61 | -1.10 | 0.00 | 0.00 |
| mir-983-2 3p | 6 | 0 | 2.44 | 0.00 | 2.71 | 0.03 | 1.00 |
| mir-927 3p | 38 | 172 | 16.57 | 69.92 | -2.25 | 0.00 | 0.00 |
| mir-986 3p | 6 | 41 | 2.44 | 17.87 | -2.69 | 0.00 | 0.00 |
| mir-6-3 3p | 80 | 68 | 34.87 | 27.64 | 0.13 | 0.58 | 1.00 |
| mir-987 3p | 3 | 7 | 1.22 | 3.05 | -1.10 | 0.20 | 1.00 |
| mir-988 3p | 731 | 618 | 318.66 | 251.23 | 0.14 | 0.07 | 1.00 |
| mir-989 3p | 26 | 133 | 10.57 | 57.98 | -2.41 | 0.00 | 0.00 |
| mir-137 3p | 283 | 525 | 123.37 | 213.42 | -0.99 | 0.00 | 0.00 |
| mir-991 3p | 28 | 0 | 11.38 | 0.00 | 4.76 | 0.00 | 0.00 |
| mir-992 3p | 4 | 0 | 1.74 | 0.00 | 2.22 | 0.10 | 1.00 |
| mir-929 3p | 26 | 41 | 10.57 | 17.87 | -0.74 | 0.04 | 1.00 |
| mir-993 3p | 411 | 394 | 179.17 | 160.17 | -0.04 | 0.69 | 1.00 |
| mir-994 3p | 0 | 2 | 0.00 | 0.87 | -1.69 | 0.28 | 1.00 |
| mir-7 3p | 572 | 912 | 249.35 | 370.75 | -0.77 | 0.00 | 0.00 |
| mir-995 3p | 1768 | 9132 | 718.73 | 3980.87 | -2.47 | 0.00 | 0.00 |
| mir-996 3p | 2644 | 4854 | 1152.59 | 1973.25 | -0.98 | 0.00 | 0.00 |
| mir-252 3p | 36 | 25 | 14.63 | 10.90 | 0.41 | 0.27 | 1.00 |
| mir-998 3p | 4685 | 4446 | 2042.31 | 1807.39 | -0.03 | 0.40 | 1.00 |
| mir-999 3p | 1498 | 0 | 608.97 | 0.00 | 10.45 | 0.00 | 0.00 |
| mir-1000 3p | 106 | 144 | 46.21 | 58.54 | -0.54 | 0.00 | 0.17 |
| mir-1001 3p | 2 | 0 | 0.81 | 0.00 | 1.48 | 0.34 | 1.00 |
| mir-1003 3p | 98 | 725 | 42.72 | 294.73 | -2.98 | 0.00 | 0.00 |
| mir-1004 3p | 31 | 5 | 12.60 | 2.18 | 2.31 | 0.00 | 0.00 |
| mir-8 3p | 199393 | 239154 | 86920.49 | 97221.18 | -0.36 | 0.00 | 0.00 |
| mir-1005 3p | 7 | 0 | 2.85 | 0.00 | 2.90 | 0.02 | 0.82 |
| mir-1006 3p | 48 | 94 | 20.92 | 38.21 | -1.06 | 0.00 | 0.00 |
| mir-1007 3p | 13 | 0 | 5.28 | 0.00 | 3.71 | 0.00 | 0.02 |
| mir-1008 3p | 178 | 0 | 77.59 | 0.00 | 7.38 | 0.00 | 0.00 |
| mir-1009 3p | 10 | 0 | 4.07 | 0.00 | 3.36 | 0.00 | 0.15 |
| mir-1011 3p | 5 | 0 | 2.18 | 0.00 | 2.48 | 0.06 | 1.00 |
| mir-1012 3p | 196 | 0 | 79.68 | 0.00 | 7.52 | 0.00 | 0.00 |
| mir-1013 3p | 87 | 0 | 37.93 | 0.00 | 6.36 | 0.00 | 0.00 |
| mir-1014 3p | 18 | 0 | 7.32 | 0.00 | 4.15 | 0.00 | 0.00 |
| mir-9a 3p | 3733 | 3441 | 1627.31 | 1398.84 | 0.02 | 0.62 | 1.00 |
| mir-1015 3p | 3 | 0 | 1.22 | 0.00 | 1.90 | 0.19 | 1.00 |
| mir-1017 3p | 41 | 32 | 17.87 | 13.01 | 0.25 | 0.46 | 1.00 |
| mir-2489 3p | 22 | 1 | 8.94 | 0.44 | 3.42 | 0.00 | 0.00 |
| mir-10 3p | 4593 | 4296 | 2002.21 | 1746.42 | 0.00 | 0.89 | 1.00 |
| mir-2493 3p | 0 | 10 | 0.00 | 4.36 | -3.56 | 0.00 | 0.07 |
| mir-2494 3p | 4 | 0 | 1.74 | 0.00 | 2.22 | 0.10 | 1.00 |
| mir-2499 3p | 1 | 0 | 0.41 | 0.00 | 0.90 | 0.60 | 1.00 |
| mir-11 3p | 14752 | 28 | 6430.77 | 11.38 | 8.89 | 0.00 | 0.00 |
| mir-2500 3p | 9 | 0 | 3.66 | 0.00 | 3.22 | 0.00 | 0.26 |
| mir-2535b 3p | 20 | 68 | 8.72 | 27.64 | -1.82 | 0.00 | 0.00 |
| mir-4919 3p | 19 | 0 | 7.72 | 0.00 | 4.22 | 0.00 | 0.00 |
| mir-13a 3p | 6363 | 11793 | 2773.79 | 4794.11 | -0.99 | 0.00 | 0.00 |
| mir-4940 3p | 1 | 0 | 0.41 | 0.00 | 0.90 | 0.60 | 1.00 |
| mir-4943 3p | 0 | 1 | 0.00 | 0.41 | -1.10 | 0.52 | 1.00 |
| mir-13b-1 3p | 66113 | 69747 | 26876.34 | 30404.50 | -0.18 | 0.00 | 0.00 |
| mir-1 3p | 420467 | 276090 | 183292.28 | 112236.45 | 0.51 | 0.00 | 0.00 |
| mir-4949 3p | 11 | 0 | 4.47 | 0.00 | 3.48 | 0.00 | 0.08 |
| mir-4955 3p | 0 | 2 | 0.00 | 0.81 | -1.69 | 0.28 | 1.00 |
| mir-4957 3p | 0 | 1 | 0.00 | 0.44 | -1.10 | 0.52 | 1.00 |
| mir-13b-2 3p | 66114 | 69748 | 28820.78 | 28354.04 | -0.18 | 0.00 | 0.00 |
| mir-4961 3p | 0 | 1 | 0.00 | 0.44 | -1.10 | 0.52 | 1.00 |
| mir-14 3p | 20688 | 25435 | 9018.43 | 10339.87 | -0.40 | 0.00 | 0.00 |
| mir-4971 3p | 0 | 1 | 0.00 | 0.44 | -1.10 | 0.52 | 1.00 |
| mir-4973 3p | 0 | 1 | 0.00 | 0.41 | -1.10 | 0.52 | 1.00 |
| mir-4977 3p | 2 | 0 | 0.81 | 0.00 | 1.48 | 0.34 | 1.00 |
| mir-263a 3p | 201 | 358 | 87.62 | 145.53 | -0.93 | 0.00 | 0.00 |
| mir-184 3p | 166328 | 79925 | 67615.87 | 34841.34 | 0.96 | 0.00 | 0.00 |
| mir-5614 3p | 0 | 7 | 0.00 | 2.85 | -3.10 | 0.01 | 0.49 |
| mir-275 3p | 10289 | 4891 | 4182.70 | 2132.11 | 0.97 | 0.00 | 0.00 |
| mir-92a 3p | 3373 | 2329 | 1470.38 | 946.79 | 0.43 | 0.00 | 0.00 |
| mir-219 3p | 34 | 32 | 13.82 | 13.95 | -0.02 | 0.96 | 1.00 |
| mir-276a 3p | 21059 | 30346 | 9180.15 | 12336.29 | -0.63 | 0.00 | 0.00 |
| mir-277 3p | 8 | 5398 | 3.25 | 2353.13 | -9.33 | 0.00 | 0.00 |
| mir-2a-1 3p | 322974 | 420122 | 140792.60 | 170788.52 | -0.48 | 0.00 | 0.00 |
| mir-278 3p | 6 | 0 | 2.44 | 0.00 | 2.71 | 0.03 | 1.00 |
| mir-133 3p | 2290 | 4494 | 998.27 | 1826.91 | -1.07 | 0.00 | 0.00 |
| mir-279 3p | 4429 | 5367 | 1800.48 | 2339.61 | -0.38 | 0.00 | 0.00 |
| mir-33 3p | 50 | 66 | 21.80 | 26.83 | -0.49 | 0.06 | 1.00 |
| mir-281-1 3p | 2179 | 2003 | 885.81 | 873.16 | 0.02 | 0.64 | 1.00 |
| mir-282 3p | 495 | 173 | 215.78 | 70.33 | 1.41 | 0.00 | 0.00 |
| mir-283 3p | 21 | 7 | 8.54 | 3.05 | 1.36 | 0.02 | 0.78 |
| mir-284 3p | 107 | 350 | 46.64 | 142.28 | -1.80 | 0.00 | 0.00 |
| mir-281-2 3p | 2179 | 2003 | 885.81 | 873.16 | 0.02 | 0.64 | 1.00 |
| mir-2a-2 3p | 468169 | 601609 | 204086.80 | 244566.84 | -0.46 | 0.00 | 0.00 |
| mir-34 3p | 133 | 68 | 54.07 | 29.64 | 0.86 | 0.00 | 0.00 |
| mir-124 3p | 6107 | 11611 | 2662.20 | 4720.12 | -1.03 | 0.00 | 0.00 |
| mir-79 3p | 0 | 2484 | 0.00 | 1082.84 | -11.38 | 0.00 | 0.00 |
| mir-276b 3p | 279 | 482 | 121.62 | 195.94 | -0.89 | 0.00 | 0.00 |
| mir-210 3p | 104 | 0 | 42.28 | 0.00 | 6.61 | 0.00 | 0.00 |
| mir-285 3p | 10 | 6 | 4.36 | 2.44 | 0.55 | 0.42 | 1.00 |
| mir-92b 3p | 6099 | 8730 | 2479.37 | 3805.63 | -0.62 | 0.00 | 0.00 |
| mir-286 3p | 194173 | 130114 | 84644.96 | 52894.11 | 0.48 | 0.00 | 0.00 |
| mir-2b-1 3p | 229943 | 196265 | 93476.72 | 85556.92 | 0.13 | 0.00 | 0.00 |
| mir-87 3p | 8962 | 4914 | 3906.76 | 1997.65 | 0.77 | 0.00 | 0.00 |
| mir-263b 3p | 8 | 4 | 3.25 | 1.74 | 0.75 | 0.34 | 1.00 |
| bantam 3p | 200549 | 141537 | 87424.42 | 57537.80 | 0.40 | 0.00 | 0.00 |
| mir-31b 3p | 5 | 0 | 2.03 | 0.00 | 2.48 | 0.06 | 1.00 |
| mir-304 3p | 389 | 495 | 169.58 | 201.23 | -0.45 | 0.00 | 0.00 |
| mir-305 3p | 141 | 181 | 57.32 | 78.90 | -0.46 | 0.00 | 0.23 |
| mir-9c 3p | 0 | 21 | 0.00 | 8.54 | -4.56 | 0.00 | 0.00 |
| mir-2b-2 3p | 221579 | 196278 | 90076.57 | 85562.58 | 0.07 | 0.00 | 0.00 |
| mir-306 3p | 0 | 23 | 0.00 | 9.35 | -4.69 | 0.00 | 0.00 |
| mir-9b 3p | 0 | 185 | 0.00 | 80.65 | -7.64 | 0.00 | 0.00 |
| let-7 3p | 2 | 0 | 0.87 | 0.00 | 1.48 | 0.34 | 1.00 |
| mir-308 3p | 148 | 124 | 60.17 | 54.05 | 0.15 | 0.38 | 1.00 |
| mir-31a 3p | 225 | 880 | 98.08 | 357.74 | -2.06 | 0.00 | 0.00 |
| mir-309 3p | 1802 | 1603 | 732.55 | 698.79 | 0.07 | 0.17 | 1.00 |
| mir-310 3p | 2318 | 152 | 1010.48 | 61.79 | 3.82 | 0.00 | 0.00 |
| mir-311 3p | 7246 | 225 | 2945.65 | 98.08 | 4.90 | 0.00 | 0.00 |
| mir-3 3p | 8673 | 13052 | 3780.78 | 5305.92 | -0.69 | 0.00 | 0.00 |
| mir-312 3p | 3143 | 0 | 1277.70 | 0.00 | 11.52 | 0.00 | 0.00 |
| mir-313 3p | 552 | 0 | 240.63 | 0.00 | 9.01 | 0.00 | 0.00 |
| mir-314 3p | 1132 | 1079 | 460.18 | 470.36 | -0.03 | 0.61 | 1.00 |
| mir-315 3p | 13 | 40 | 5.67 | 16.26 | -1.65 | 0.00 | 0.00 |
| mir-316 3p | 30 | 62 | 12.20 | 27.03 | -1.12 | 0.00 | 0.01 |
| mir-317 3p | 153 | 348 | 66.70 | 141.47 | -1.28 | 0.00 | 0.00 |
| mir-318 3p | 9 | 12 | 3.66 | 5.23 | -0.48 | 0.43 | 1.00 |
| mir-2c 3p | 5318 | 7386 | 2318.25 | 3002.57 | -0.57 | 0.00 | 0.00 |
| mir-iab-4 3p | 9 | 9 | 3.66 | 3.92 | -0.10 | 0.88 | 1.00 |
| mir-4 3p | 8933 | 8807 | 3894.12 | 3580.23 | -0.08 | 0.00 | 0.01 |
| mir-955 3p | 23 | 0 | 9.35 | 0.00 | 4.48 | 0.00 | 0.00 |
| mir-190 3p | 663 | 367 | 289.02 | 149.19 | 0.75 | 0.00 | 0.00 |
| mir-956 3p | 385 | 618 | 156.51 | 269.40 | -0.78 | 0.00 | 0.00 |
| mir-957 3p | 140 | 120 | 61.03 | 48.78 | 0.12 | 0.50 | 1.00 |
| mir-958 3p | 676 | 1276 | 274.81 | 556.24 | -1.02 | 0.00 | 0.00 |
| mir-375 3p | 672 | 1218 | 292.94 | 495.14 | -0.96 | 0.00 | 0.00 |
| mir-959 3p | 2 | 0 | 0.81 | 0.00 | 1.48 | 0.34 | 1.00 |
| mir-5 3p | 4818 | 6102 | 2100.29 | 2480.59 | -0.44 | 0.00 | 0.00 |
| mir-964 3p | 3 | 0 | 1.22 | 0.00 | 1.90 | 0.19 | 1.00 |
| mir-932 3p | 45 | 53 | 19.62 | 21.55 | -0.33 | 0.25 | 1.00 |
| mir-965 3p | 324 | 533 | 131.71 | 232.35 | -0.82 | 0.00 | 0.00 |
| mir-967 3p | 6 | 0 | 2.62 | 0.00 | 2.71 | 0.03 | 1.00 |
| mir-1002 3p | 167 | 0 | 67.89 | 0.00 | 7.29 | 0.00 | 0.00 |
| mir-968 3p | 385 | 44 | 167.83 | 17.89 | 3.00 | 0.00 | 0.00 |
| mir-6-1 3p | 70 | 64 | 28.46 | 27.90 | 0.03 | 0.91 | 1.00 |
| mir-969 5p | 1 | 0 | 0.41 | 0.00 | 0.90 | 0.60 | 1.00 |
| mir-970 5p | 34 | 57 | 14.82 | 24.85 | -0.83 | 0.01 | 0.35 |
| mir-971 5p | 0 | 16 | 0.00 | 6.50 | -4.19 | 0.00 | 0.00 |
| mir-6-2 5p | 77 | 39 | 33.57 | 17.00 | 0.86 | 0.00 | 0.10 |
| mir-980 5p | 49 | 22 | 19.92 | 8.94 | 1.02 | 0.00 | 0.21 |
| mir-981 5p | 122 | 185 | 53.18 | 80.65 | -0.70 | 0.00 | 0.00 |
| mir-982 5p | 13 | 0 | 5.28 | 0.00 | 3.71 | 0.00 | 0.03 |
| mir-983-2 5p | 16 | 0 | 6.97 | 0.00 | 3.99 | 0.00 | 0.00 |
| mir-927 5p | 199 | 328 | 80.90 | 133.34 | -0.82 | 0.00 | 0.00 |
| mir-986 5p | 579 | 947 | 252.40 | 412.82 | -0.81 | 0.00 | 0.00 |
| mir-6-3 5p | 117 | 184 | 47.56 | 74.80 | -0.75 | 0.00 | 0.00 |
| mir-987 5p | 791 | 834 | 344.82 | 363.56 | -0.18 | 0.01 | 0.72 |
| mir-988 5p | 59 | 53 | 23.98 | 21.55 | 0.05 | 0.85 | 1.00 |
| mir-137 5p | 12 | 108 | 5.23 | 47.08 | -3.17 | 0.00 | 0.00 |
| mir-990 5p | 12 | 2 | 4.88 | 0.81 | 2.01 | 0.01 | 0.73 |
| mir-992 5p | 3 | 0 | 1.31 | 0.00 | 1.90 | 0.19 | 1.00 |
| mir-929 5p | 1366 | 1646 | 555.31 | 669.13 | -0.37 | 0.00 | 0.00 |
| mir-993 5p | 28 | 46 | 12.21 | 20.05 | -0.80 | 0.02 | 0.91 |
| mir-7 5p | 5346 | 3137 | 2173.26 | 1275.26 | 0.67 | 0.00 | 0.00 |
| mir-995 5p | 93 | 143 | 40.54 | 62.34 | -0.72 | 0.00 | 0.01 |
| mir-996 5p | 817 | 1221 | 332.13 | 496.36 | -0.68 | 0.00 | 0.00 |
| mir-252 5p | 5593 | 9441 | 2438.13 | 4115.57 | -0.86 | 0.00 | 0.00 |
| mir-998 5p | 70 | 105 | 28.46 | 42.68 | -0.68 | 0.00 | 0.12 |
| mir-999 5p | 9 | 4102 | 3.92 | 1788.17 | -8.78 | 0.00 | 0.00 |
| mir-1000 5p | 6554 | 11532 | 2664.34 | 4688.00 | -0.92 | 0.00 | 0.00 |
| mir-1001 5p | 11 | 0 | 4.80 | 0.00 | 3.48 | 0.00 | 0.09 |
| mir-1003 5p | 37 | 38 | 15.04 | 15.45 | -0.14 | 0.67 | 1.00 |
| mir-1004 5p | 22 | 1 | 9.59 | 0.44 | 3.42 | 0.00 | 0.00 |
| mir-8 5p | 7979 | 7203 | 3243.63 | 2928.17 | 0.05 | 0.05 | 1.00 |
| mir-1005 5p | 1 | 0 | 0.44 | 0.00 | 0.90 | 0.60 | 1.00 |
| mir-1006 5p | 12 | 12 | 4.88 | 4.88 | -0.10 | 0.86 | 1.00 |
| mir-1007 5p | 12 | 0 | 5.23 | 0.00 | 3.60 | 0.00 | 0.05 |
| mir-1008 5p | 3 | 0 | 1.22 | 0.00 | 1.90 | 0.19 | 1.00 |
| mir-1009 5p | 4 | 0 | 1.74 | 0.00 | 2.22 | 0.10 | 1.00 |
| mir-1010 5p | 2417 | 2357 | 982.56 | 958.17 | -0.06 | 0.12 | 1.00 |
| mir-1012 5p | 236 | 0 | 102.88 | 0.00 | 7.79 | 0.00 | 0.00 |
| mir-1014 5p | 19 | 3 | 7.72 | 1.22 | 2.22 | 0.00 | 0.08 |
| mir-9a 5p | 85999 | 71136 | 37489.16 | 31010.00 | 0.17 | 0.00 | 0.00 |
| mir-1015 5p | 2 | 0 | 0.81 | 0.00 | 1.48 | 0.34 | 1.00 |
| mir-1017 5p | 1 | 0 | 0.44 | 0.00 | 0.90 | 0.60 | 1.00 |
| mir-2282 5p | 0 | 28 | 0.00 | 11.38 | -4.96 | 0.00 | 0.00 |
| mir-10 5p | 29631 | 21018 | 12916.91 | 9162.28 | 0.39 | 0.00 | 0.00 |
| mir-2493 5p | 0 | 5 | 0.00 | 2.03 | -2.69 | 0.04 | 1.00 |
| mir-2494 5p | 4 | 0 | 1.74 | 0.00 | 2.22 | 0.10 | 1.00 |
| mir-2498 5p | 1 | 0 | 0.41 | 0.00 | 0.90 | 0.60 | 1.00 |
| mir-11 5p | 231 | 24474 | 100.70 | 10668.84 | -6.82 | 0.00 | 0.00 |
| mir-2500 5p | 14 | 0 | 5.69 | 0.00 | 3.81 | 0.00 | 0.01 |
| mir-3642 5p | 0 | 1 | 0.00 | 0.44 | -1.10 | 0.52 | 1.00 |
| mir-12 5p | 4602 | 9260 | 1870.81 | 3764.39 | -1.11 | 0.00 | 0.00 |
| mir-2535b 5p | 8 | 10 | 3.49 | 4.36 | -0.39 | 0.55 | 1.00 |
| mir-4919 5p | 9 | 0 | 3.66 | 0.00 | 3.22 | 0.00 | 0.28 |
| mir-13a 5p | 108 | 247 | 47.08 | 107.67 | -1.29 | 0.00 | 0.00 |
| mir-13b-1 5p | 41 | 84 | 16.67 | 34.15 | -1.12 | 0.00 | 0.00 |
| mir-1 5p | 9 | 73 | 3.92 | 31.82 | -2.99 | 0.00 | 0.00 |
| mir-4949 5p | 1 | 1 | 0.41 | 0.41 | -0.10 | 0.94 | 1.00 |
| mir-4951 5p | 2 | 2 | 0.87 | 0.87 | -0.10 | 0.93 | 1.00 |
| mir-4956 5p | 0 | 3 | 0.00 | 1.22 | -2.10 | 0.14 | 1.00 |
| mir-4957 5p | 1 | 0 | 0.44 | 0.00 | 0.90 | 0.60 | 1.00 |
| mir-13b-2 5p | 53 | 53 | 21.55 | 21.55 | -0.10 | 0.72 | 1.00 |
| mir-4962 5p | 0 | 9 | 0.00 | 3.92 | -3.42 | 0.00 | 0.15 |
| mir-4963 5p | 1 | 0 | 0.41 | 0.00 | 0.90 | 0.60 | 1.00 |
| mir-4968 5p | 3 | 0 | 1.31 | 0.00 | 1.90 | 0.19 | 1.00 |
| mir-14 5p | 902 | 825 | 366.68 | 335.38 | 0.03 | 0.69 | 1.00 |
| mir-4974 5p | 1 | 0 | 0.44 | 0.00 | 0.90 | 0.60 | 1.00 |
| mir-4976 5p | 1 | 0 | 0.41 | 0.00 | 0.90 | 0.60 | 1.00 |
| mir-263a 5p | 34326 | 22970 | 14963.58 | 10013.21 | 0.48 | 0.00 | 0.00 |
| mir-4985 5p | 0 | 1 | 0.00 | 0.41 | -1.10 | 0.52 | 1.00 |
| mir-4987 5p | 1 | 1 | 0.44 | 0.44 | -0.10 | 0.94 | 1.00 |
| mir-184 5p | 81 | 95 | 32.93 | 38.62 | -0.33 | 0.13 | 1.00 |
| mir-5614 5p | 0 | 29 | 0.00 | 12.64 | -5.01 | 0.00 | 0.00 |
| mir-274 5p | 0 | 10 | 0.00 | 4.07 | -3.56 | 0.00 | 0.08 |
| mir-275 5p | 77 | 31 | 33.57 | 13.51 | 1.18 | 0.00 | 0.00 |
| mir-92a 5p | 1671 | 1473 | 679.30 | 598.81 | 0.08 | 0.12 | 1.00 |
| mir-219 5p | 2 | 6 | 0.87 | 2.62 | -1.32 | 0.16 | 1.00 |
| mir-276a 5p | 24601 | 19797 | 10000.83 | 8047.90 | 0.21 | 0.00 | 0.00 |
| mir-277 5p | 508 | 857 | 221.45 | 373.59 | -0.85 | 0.00 | 0.00 |
| mir-2a-1 5p | 249 | 65 | 101.22 | 26.42 | 1.82 | 0.00 | 0.00 |
| mir-278 5p | 77 | 77 | 33.57 | 33.57 | -0.10 | 0.66 | 1.00 |
| mir-133 5p | 434 | 343 | 176.43 | 139.44 | 0.24 | 0.02 | 1.00 |
| mir-279 5p | 360 | 580 | 156.93 | 252.84 | -0.79 | 0.00 | 0.00 |
| mir-33 5p | 1778 | 1419 | 722.79 | 576.85 | 0.22 | 0.00 | 0.00 |
| mir-280 5p | 0 | 5 | 0.00 | 2.18 | -2.69 | 0.04 | 1.00 |
| mir-281-1 5p | 2459 | 1360 | 999.64 | 552.87 | 0.75 | 0.00 | 0.00 |
| mir-282 5p | 1413 | 2548 | 615.96 | 1110.74 | -0.95 | 0.00 | 0.00 |
| mir-283 5p | 9127 | 5132 | 3710.32 | 2086.27 | 0.73 | 0.00 | 0.00 |
| mir-284 5p | 55 | 236 | 23.98 | 102.88 | -2.18 | 0.00 | 0.00 |
| mir-281-2 5p | 2459 | 1386 | 999.64 | 563.44 | 0.73 | 0.00 | 0.00 |
| mir-2a-2 5p | 755 | 1924 | 329.12 | 838.72 | -1.45 | 0.00 | 0.00 |
| mir-34 5p | 1571 | 516 | 638.64 | 209.76 | 1.50 | 0.00 | 0.00 |
| mir-124 5p | 7 | 21 | 3.05 | 9.15 | -1.56 | 0.01 | 0.29 |
| mir-79 5p | 0 | 206 | 0.00 | 83.74 | -7.79 | 0.00 | 0.00 |
| mir-276b 5p | 24601 | 19796 | 10724.20 | 8629.58 | 0.21 | 0.00 | 0.00 |
| mir-210 5p | 5 | 97 | 2.03 | 39.43 | -4.13 | 0.00 | 0.00 |
| mir-285 5p | 3 | 2 | 1.31 | 0.87 | 0.31 | 0.77 | 1.00 |
| mir-100 5p | 2 | 1 | 0.81 | 0.41 | 0.48 | 0.71 | 1.00 |
| mir-92b 5p | 131 | 151 | 57.11 | 65.82 | -0.30 | 0.08 | 1.00 |
| mir-286 5p | 40 | 418 | 16.26 | 169.93 | -3.45 | 0.00 | 0.00 |
| mir-2b-1 5p | 3 | 88 | 1.31 | 38.36 | -4.58 | 0.00 | 0.00 |
| mir-87 5p | 27 | 14 | 10.98 | 5.69 | 0.80 | 0.08 | 1.00 |
| mir-263b 5p | 4893 | 764 | 2132.98 | 333.05 | 2.58 | 0.00 | 0.00 |
| bantam 5p | 3302 | 2654 | 1342.33 | 1078.91 | 0.21 | 0.00 | 0.00 |
| mir-303 5p | 4 | 0 | 1.74 | 0.00 | 2.22 | 0.10 | 1.00 |
| mir-31b 5p | 785 | 719 | 319.12 | 292.29 | 0.03 | 0.73 | 1.00 |
| mir-304 5p | 754 | 893 | 328.69 | 389.28 | -0.34 | 0.00 | 0.00 |
| mir-305 5p | 0 | 3823 | 0.00 | 1554.13 | -12.00 | 0.00 | 0.00 |
| mir-9c 5p | 0 | 11037 | 0.00 | 4811.31 | -13.53 | 0.00 | 0.00 |
| mir-2b-2 5p | 1341 | 798 | 545.14 | 324.40 | 0.65 | 0.00 | 0.00 |
| mir-306 5p | 0 | 6463 | 0.00 | 2817.39 | -12.76 | 0.00 | 0.00 |
| mir-9b 5p | 0 | 15747 | 0.00 | 6401.49 | -14.04 | 0.00 | 0.00 |
| let-7 5p | 70 | 66 | 30.51 | 28.77 | -0.02 | 0.94 | 1.00 |
| mir-125 5p | 26 | 16 | 10.57 | 6.50 | 0.57 | 0.20 | 1.00 |
| mir-308 5p | 885 | 602 | 385.79 | 262.43 | 0.45 | 0.00 | 0.00 |
| mir-31a 5p | 19645 | 23278 | 7986.11 | 9463.00 | -0.35 | 0.00 | 0.00 |
| mir-309 5p | 30 | 7 | 13.08 | 3.05 | 1.85 | 0.00 | 0.02 |
| mir-310 5p | 18 | 52 | 7.32 | 21.14 | -1.58 | 0.00 | 0.00 |
| mir-311 5p | 130 | 0 | 56.67 | 0.00 | 6.93 | 0.00 | 0.00 |
| mir-3 5p | 9 | 40 | 3.66 | 16.26 | -2.14 | 0.00 | 0.00 |
| mir-312 5p | 213 | 1032 | 92.85 | 449.88 | -2.37 | 0.00 | 0.00 |
| mir-313 5p | 228 | 0 | 92.69 | 0.00 | 7.74 | 0.00 | 0.00 |
| mir-314 5p | 51 | 103 | 22.23 | 44.90 | -1.10 | 0.00 | 0.00 |
| mir-315 5p | 12279 | 9802 | 4991.67 | 3984.72 | 0.22 | 0.00 | 0.00 |
| mir-316 5p | 3119 | 4410 | 1359.65 | 1922.43 | -0.60 | 0.00 | 0.00 |
| mir-317 5p | 20 | 27 | 8.13 | 10.98 | -0.52 | 0.21 | 1.00 |
| mir-2c 5p | 55 | 13 | 23.98 | 5.67 | 1.90 | 0.00 | 0.00 |
| mir-iab-4 5p | 176 | 326 | 71.55 | 132.53 | -0.99 | 0.00 | 0.00 |
| mir-4 5p | 182 | 503 | 79.34 | 219.27 | -1.56 | 0.00 | 0.00 |
| mir-955 5p | 645 | 0 | 262.21 | 0.00 | 9.23 | 0.00 | 0.00 |
| mir-190 5p | 328 | 191 | 142.98 | 83.26 | 0.68 | 0.00 | 0.00 |
| mir-193 5p | 0 | 1 | 0.00 | 0.41 | -1.10 | 0.52 | 1.00 |
| mir-956 5p | 4 | 7 | 1.74 | 3.05 | -0.78 | 0.34 | 1.00 |
| mir-957 5p | 3 | 8 | 1.22 | 3.25 | -1.27 | 0.13 | 1.00 |
| mir-958 5p | 3461 | 2875 | 1508.74 | 1253.29 | 0.17 | 0.00 | 0.00 |
| mir-375 5p | 460 | 54 | 187.00 | 21.95 | 2.97 | 0.00 | 0.00 |
| mir-959 5p | 0 | 3 | 0.00 | 1.31 | -2.10 | 0.14 | 1.00 |
| mir-960 5p | 2 | 0 | 0.81 | 0.00 | 1.48 | 0.34 | 1.00 |
| mir-5 5p | 9235 | 11257 | 4025.77 | 4907.21 | -0.39 | 0.00 | 0.00 |
| mir-961 5p | 4 | 0 | 1.63 | 0.00 | 2.22 | 0.10 | 1.00 |
| mir-962 5p | 10 | 0 | 4.36 | 0.00 | 3.36 | 0.00 | 0.15 |
| mir-963 5p | 21 | 0 | 8.54 | 0.00 | 4.36 | 0.00 | 0.00 |
| mir-964 5p | 5 | 1 | 2.18 | 0.44 | 1.48 | 0.18 | 1.00 |
| mir-932 5p | 184 | 496 | 74.80 | 201.63 | -1.53 | 0.00 | 0.00 |
| mir-965 5p | 472 | 118 | 205.76 | 51.44 | 1.89 | 0.00 | 0.00 |
| mir-967 5p | 16 | 1 | 6.50 | 0.41 | 2.99 | 0.00 | 0.03 |
| mir-1002 5p | 5686 | 356 | 2478.67 | 155.19 | 3.89 | 0.00 | 0.00 |
| mir-968 5p | 28624 | 35340 | 11636.26 | 14366.46 | -0.40 | 0.00 | 0.00 |
| mir-6-1 5p | 29 | 1267 | 12.64 | 552.32 | -5.50 | 0.00 | 0.00 |

## Supplementary Figure Legends:

## Figure S1 - The miRNA prediction interface of miREvo.

The default parameter settings were shown.

## Figure S2 - Othologs of members of ath-miR-169 family across 11 green plant genomes.

[Included separately as Additional file 2]

## Supplementary Figures:

## Figure S1


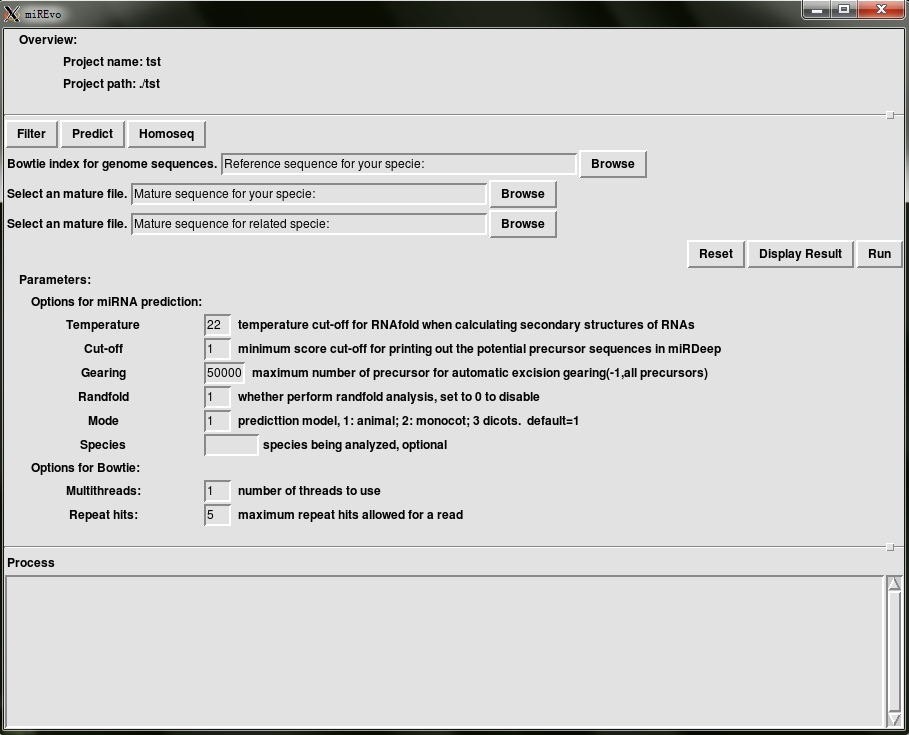


## Figure S2

[Included separately as Additional file 2]
