# Supplementary material for: miREvo: an integrative microRNA evolutionary analysis platform for next-generation sequencing experiments
Source: BMC Bioinformatics. 2012 Jun 21;13:140. doi: 10.1186/1471-2105-13-140 (PMC3410788; doi:10.1186/1471-2105-13-140)
Supplement: Additional file 2 — Plant Parameters.doc. The modification of miRDeep2 parameters that are used for the prediction of novel plant miRNAs in miREvo. [file 1471-2105-13-140-S2.doc]

Notes:

1. The changes specified here are in two of the PERL scripts of mirdeep2_0_0_3.zip, downloaded on Nov 2011.
2. The syntax to be changed has been referred by line numbers. Changes should preferably be made in decreasing order of line numbers.
3. For convenience, both the syntax to be replaced (labelled with '<') and its replacement (labelled with '>'), have been explicitly mentioned. Make sure to REMOVE THERE LABELS from the PERL scripts.

===========================================

**ADD PROGRAM: excise_precursors_iterative_plant.pl, modified from excise_precursors_iterative_final.pl**

===========================================

**TOTAL 4 CHANGES:**

===========================================

1. **Add two lines #33-34:**

**Explanation: Increase the excision length, new parameters provided for plant.**

> my $plant_up=22;

> my $plant_down=276;

______________________________________________________

2. **REPLACE line #283-284:**

**Explanation: To adjust the maximum excision length**

< my $excise_beg=$db_beg-70;

< my $excise_end=$db_end+20;

---**-------By-------**

> my $excise_beg=$db_beg-$plant_down;

> my $excise_end=$db_end+$plant_up;

____________________________________________________

3 **REPLACE line #290-291:**

**Explanation: To adjust the maximum excision length**

< $excise_beg=$db_beg-20;

< $excise_end=$db_end+70;

---**-------By-------**

**> $excise_beg=$db_beg-$plant_up;**

**> $excise_end=$db_end+$plant_down;**

____________________________________________________

4. **REPLACE line #335:**

**Explanation: To adjust the maximum excision length**

< for(my $pos_beg=$db_beg+1; $pos_beg<=$db_end+70; $pos_beg++){

---**-------By-------**

**> for(my $pos_beg=$db_beg+1; $pos_beg<=$db_end+$plant_down; $pos_beg++){**

____________________________________________________

===========================================

**ADD PROGRAM: miRDeep2_core_plant.pl, modified from miRDeep2_core_algorithm.pl**

===========================================

**TOTAL 10 CHANGES**

===========================================

1. **Add one line #37:**

**Explanation: New program options provided for plant miRNA prediction.**

> -d set parameters for dicots miRNA prediction, default is for monocot

____________________________________________________

2. **REPLACE #57:**

**Explanation: New program options provided for plant miRNA prediction.**

< getopts("hs:tuv:xy:z",\%options);

---**-------By-------**

> getopts("hs:tuv:xy:zd",\%options);

____________________________________________________

3. **COMMENT lines #1680-1703 (Put '#' in the beginning of specified lines):**

**Explanation: Two subroutines, prob_gumbel_discretized() and cdf_gumbel(), are no longer required**

______________________________________________________

4. **REPLACE lines #1663-1673:**

**Explanation: Since the MFE scoring here uses log-odds, so the subroutine score_mfe() needs to be modified**

< my $mfe=shift;

< #numerical value, minimum 1

< my $mfe_adj=max2(1,-$mfe);

< #parameters of known precursors and background hairpins, scale and location

< my $prob_test=prob_gumbel_discretized($mfe_adj,5.5,32);

< my $prob_background=prob_gumbel_discretized($mfe_adj,4.8,23);

< my $odds=$prob_test/$prob_background;

< my $log_odds=log($odds);

**-------By-------**

> my ($mfe,$pri_lng)=@_;

> #normalize the MFE by length

> my $mfe_adj=$mfe/$pri_lng;

> #instead of finding individual functions for real and bgr, the one for log-odds was directly obtained.

> #Its a sigmoid func. with +ve x. f(x)=a/(b+exp(x*c))

> my $param_a=1.339e-12; my $param_b= 2.77826e-13; my $pa:ram_c= 45.8426; my $log_odds=0;

> my $param_a=1.339e-12; my $param_b= 2.77826e-13; my $param_c= 45.8426; my $log_odds=0;

> #dicots

> if($options{d}){

> $param_a=4.46e-4; $param_b= 9.125e-5; $param_c= 26.9293;

> }

> $log_odds=$param_a/($param_b+exp($mfe_adj*$param_c));

5. **REPLACE #573:**

**Explanation: Since duplex properties are different so modify/add filters of subroutine 'pass_filtering_structure()'**

< unless(no_bifurcations_precursor()){$ret=0;}

**-------By-------**

> # unless(no_bifurcations_precursor()){$ret=0;}

> #not more than 3 (defualt=5) nt difference between mature and star length

> unless(-3<=diff_lng() and diff_lng()<=3) {$ret=0; filter_s("too large difference between mature and star length"); }

> #maximum 5 unpaired bases

> unless (ttl_unpaired()<6) {$ret=0; filter_s("too many unpaired bases in duplex");}

> #maximum 3 consecutive unpaired bases

> unless (consc_unpaired()<=3) {$ret=0; filter_s("too long size of consecutive unpaired bases in duplex");}

____________________________________________________

6. **ADD TWO SUBROUTINES TO THE END:**

**Explanation: Since filters were added to pass_filtering_structure(), new subroutines are now required- i) ttl_unpaired()', ii) 'consc_unpaired()'**

> sub ttl_unpaired{

> my $mature_struct=$hash_comp{"mature_struct"};

> #total unpaired

> my $count_unpaired=$mature_struct=~s/\./\./g;

> return $count_unpaired;

> }

> sub consc_unpaired{

> my $mature_struct=$hash_comp{"mature_struct"};

> #replace first the paired by '1', and split the string based on '1'

> $mature_struct=~s/\(+/1/g; $mature_struct=~s/\)+/1/g;

> my @unprd=split '1',$mature_struct;

> my @list_unprd="";

> shift @list_unprd;

> #count them and store in an array

> my $elem=""; my $num_unprd="";

> foreach $elem(@unprd) {

> if ($elem=~/\./g) {$num_unprd=$elem=~s/\./\./g;push @list_unprd,$num_unprd;}

> }

> #if the array is not NULL, then SORT the consc_unprd in ASCENDING order

> if ($#list_unprd>=0){

> @list_unprd= (sort {$a<=>$b} @list_unprd);

> return pop(@list_unprd);

> }

> else {return 0;}

> }

______________________________________________________

7. **REPLACE line #259:**

**Explanation: One aditional argument passed in subroutine 'score_mfe()'**

< my $score_mfe=score_mfe($hash_comp{"pri_mfe"});

**-------By-------**

> my $score_mfe=score_mfe($hash_comp{"pri_mfe"},$hash_comp{"pri_end"});

_____________________________________________________

8. **REPLACE lines #70:**

**Explanation: Provide new parameters**

< my $seed_lng=7;

**-------By-------**

> my $seed_lng =11; #new=11 (2..12) Default=7

_____________________________________________________

9. **REPLACE lines #75-78:**

**Explanation: Provide new parameters**

< my $score_seed=3;

< my $score_seed_not=-0.6;

< my $score_randfold=1.6;

< my $score_randfold_not=-2.2;

**-------By-------**

> my $score_seed=7.635;

> my $score_seed_not=-1.17;

_____________________________________________________

10. **add following lines at #83:**

**Explanation: Provide new parameters**

> my $score_randfold=1.37;

> my $score_randfold_not=-3.624;

> if($options{d}){

> $score_randfold=0.631;

> $score_randfold_not=-3.1749;

> }

_____________________________________________________
